# Visceral Leishmaniasis: New Health Tools Are Needed

**DOI:** 10.1371/journal.pmed.0020211

**Published:** 2005-07-26

**Authors:** Asrat Hailu, Ahmed Mudawi Musa, Catherine Royce, Monique Wasunna

## Abstract

Half a million new cases of visceral leishmaniasis occur each year, and 10% of these are fatal. New tools are urgently needed for mapping, diagnosing, and treating the disease.

Visceral leishmaniasis (VL), commonly known as kala-azar, from the Hindu vernacular, is a human systemic disease caused by parasitic protozoan species of the genus *Leishmania*. Transmitted by the bite of the tiny and seemingly innocuous female phlebotomine sandfly (Figure 1), the parasite enters macrophages, where it multiplies and establishes the infection (Figure 2).

**Figure 1:**
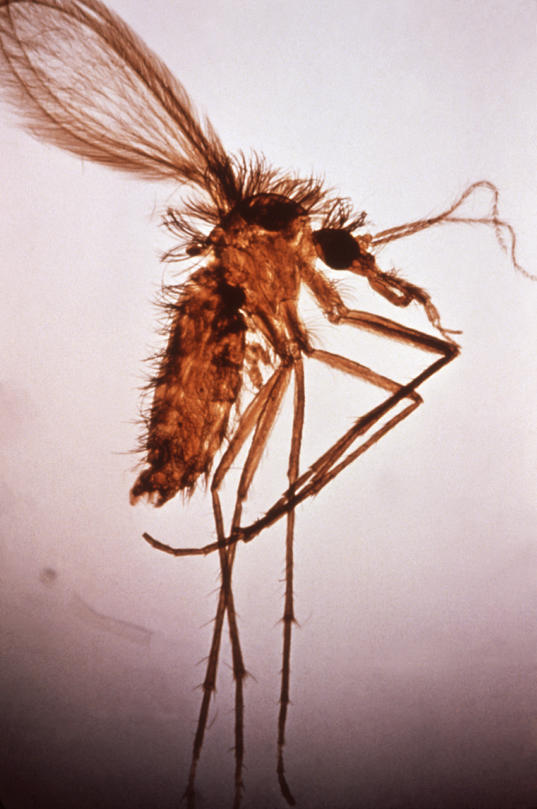
Female *Phlebotomus* sp. Sandfly (Photo: WHO/CDC)

**Figure 2:**
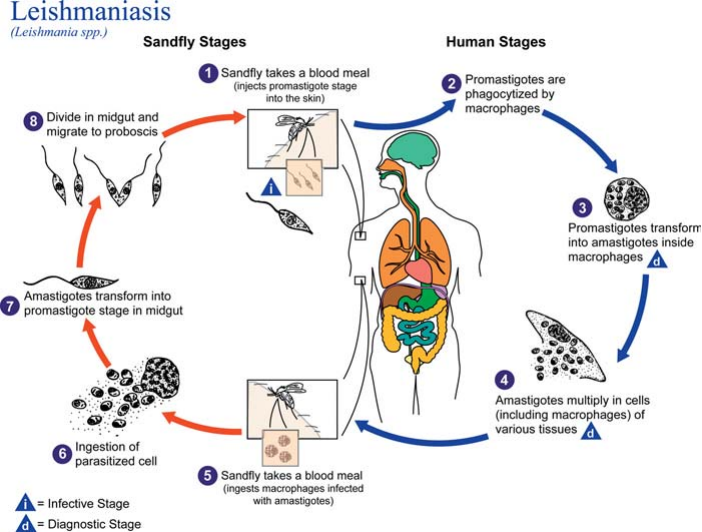
The Life Cycle of *Leishmania* spp., the Causal Agents of Leishmaniasis Leishmaniasis is transmitted by the bite of female phlebotomine sandflies. The sandflies inject the infective stage, promastigotes, during blood meals (1). Promastigotes that reach the puncture wound are phagocytized by macrophages (2) and transform into amastigotes (3). Amastigotes multiply in infected cells and affect different tissues, depending in part on the *Leishmania* species (4). This originates the clinical manifestations of leishmaniasis. Sandflies become infected during blood meals on an infected host when they ingest macrophages infected with amastigotes (5 and 6). In the sandfly's midgut, the parasites differentiate into promastigotes (7), which multiply and migrate to the proboscis (8). (Illustration: CDC/Alexander J da Silva/Melanie Moser)

A multitude of clinical features of the disease ensue gradually, the most important being splenomegaly, recurring and irregular fever, anaemia, pancytopenia, weight loss, and weakness. Unlike malaria, there is no early dramatic fever to announce its arrival; the presentation is insidious, with symptoms appearing over a period of weeks or even months. Affected patients become progressively more anaemic, weak, cachectic, and susceptible to intercurrent infections. The disease is a silent killer, invariably killing almost all untreated patients [1]. VL affects not only the weakest in the community, such as children and those weakened by other diseases such as HIV and tuberculosis, but also healthy adults and economically productive social groups.

The disease is a silent killer, invariably killing almost all untreated patients

An estimated 500,000 new cases of VL occur each year, and a tenth of these patients will die [2]. The actual death toll from the disease may be higher than this estimate, considering the existence of unidentified VL foci.

Some 90% of those affected by the disease live in five countries: India (especially Bihar), Bangladesh, Nepal, (northeastern) Brazil, and Sudan [2]. VL often exists in areas that are either remote or not easily accessible, and where health facilities are barely available or inadequate. Those most likely to be infected are people who are poor, living in villages far from roads and health-care centres. Patients from such remote communities often die in the villages without seeking treatment. Some may attempt to report to distant health-care centres, but in many cases it is simply too late. Even if they can make the journey to a hospital, they would still succumb to the illness because of the absence of anti-leishmanial drugs. Thus, many decide to stay at home until they die. But in doing so, they act as a reservoir of infection, passing on the parasite to family and neighbours through the bite of sandflies.

At present, approaches to the control of VL are varied. This variety is dictated first and foremost by the diverse epidemiological patterns of the disease, which range from domestic zoonosis (see Glossary) involving the dog (the Mediterranean littoral) or sylvatic zoonosis (South America, and possibly Africa) to anthroponosis (India and Africa). The major epidemics of VL that have occurred in India and Africa have primarily been a result of human-to-human transmission, be it in a primarily anthroponotic or zoonotic focus. Knowledge of the epidemiology, and ecological types, of VL is of paramount importance in designing a sound VL control strategy.

## Identifying Patients and Mapping the Distribution: The Need for Improved Tools

Most VL infections occur in remote geographical areas where health facilities are not well established and where the infections often co-exist with malaria and other debilitating parasitic infections. Under these circumstances, the disease usually presents a diagnostic dilemma.

To alleviate this difficulty, health workers need to be provided with up-to-date information on the geographical distribution of VL in endemic countries. The mapping of VL is a complex undertaking, as the distribution of the disease is multifocal in nature, with remarkable variation in its prevalence and incidence. Moreover, most clinical cases are neither treated nor reported. This difficulty is further compounded by the fact that most infections of *Leishmania* are subclinical.

The main activities of mapping involve active surveillance by case-finding, leishmanin skin test surveys, and serological screening of populations. Effective mapping activities will also require additional information about the sandfly. As capacities for undertaking such surveys are limited, mapping activities may be based on the use of the Geographic Information System (GIS), by which risk areas of VL can be predicted in areas where surveillance data are not readily available [3,4]. Using GIS, it is possible to produce predicted-risk maps of the disease, based on statistical associations between the spatially comprehensive environmental data available from satellites and previous knowledge about disease distribution. GIS has been used to produce risk maps of VL in some countries, and it is hoped that the maps will assist in planning future vector-control programmes.

The leishmanin skin test has been used for geographical mapping and for epidemiological description of transmission patterns [5–7]. However, its large-scale use has been frustrated by the absence of a standardised test antigen and models for interpretation of field data. Periodic active case-finding surveys are often used in mapping activities. But the labour-intensive nature of the endeavour does not permit wide coverage. The development of simple and robust screening techniques, such as the fast agglutination screening test (FAST), can be a useful addition for mapping activities [8]. Using FAST, large numbers of blood samples can be tested in a short time and can provide an indicator of past or present disease or infection. Field experiences with FAST have indicated the need for further improvements of the test with respect to robustness as well as cost. Other test systems for use in epidemiological mapping are yet to be developed.

## Estimates of Incidence

Incidence of VL varies from place to place depending on the epidemiological characteristics. Areas of sporadic endemicity (low incidence) and endemo-epidemicity (high incidence) are known to exist. High incidence rates of VL are common in areas where human populations are despoiled by social instability, war, and migration. However, accurate data on the burden of VL do not exist in many VL foci, as large proportions of VL cases are not recorded. Researchers and clinicians working in the field estimate that in some countries less than 20% of patients are currently being reached, even though this may vary from country to country.

The failure of governments to reach patients is one of the main reasons for the increasing death toll and the ever-increasing incidence of the disease. Control strategies for VL need to highlight the importance of treatment not only to reduce morbidity and mortality but also to prevent the accumulation of cases. These strategies require the availability of simple diagnostic tools and affordable, easy-to-administer drugs.

## Conventional Diagnostics: Invasive and Potentially Dangerous

Demonstration of parasites in stained smears of tissue aspirates from spleen, bone marrow, or lymph node remains the most accurate (specific) method available for diagnosis of VL. Spleen and bone marrow are both superior to lymph node but more invasive. Obtaining aspirates from the spleen can be dangerous in patients with haematological complications. Culture of the *Leishmania* parasite from tissue aspirates in Novy-MacNeal-Nicolle or Schneider's insect medium supplemented with 10% v/v foetal calf serum, if properly performed, is a more sensitive technique.

Serological tests based on the detection of specific humoral antibodies are less invasive. Such tests include indirect fluorescent antibody test, direct agglutination test (DAT), enzyme-linked immunosorbent assay, and rK39 dipstick test [9,10]. However, these tests, with the exception of the last, require trained personnel and considerable laboratory facilities. False positives can occur when these tests are used. Furthermore, serological tests may remain positive after successful treatment and give false negative results in patients with VL and HIV co-infection. Nonetheless, serological tests have been used in screening patients (to exclude other causes of febrile hepatosplenomegaly) and to support clinical diagnosis of VL.

Control programmes for VL in some countries use diagnostic algorithms that also include DAT and the dipstick test systems. DAT has been used widely under field conditions. The existing dipstick test systems (such as rK39) are attractive options. Preliminary field trials of rK39 have shown promising but geographically variable results. Together with DAT, the rK39 dipstick test and urine antigen detection tests are currently under evaluation by the World Health Organization in different countries. The development of a sensitive, specific, simple, and affordable test for use in field settings will be a crucial step in the control of VL. Thus, the need to improve rK39, and the development of new dipstick systems, is vital.

## Current Treatments: Old, Toxic, and Difficult to Deliver

For many decades, the treatment of VL has been based on pentavalent antimonials, such as sodium stibogluconate (Pentostam) or meglumine antimoniate (Glucantime), given intramuscularly or intravenously for one month.

Discovered 60 years ago, sodium stibogluconate remains the mainstay treatment of VL despite its cardiotoxicity in some patients. Treatment requires 30 days of intramuscular or intraveneous injections in a hospital setting. Although it is still effective in most endemic countries, with 95% cure rates, resistance is increasing in some regions, especially in northern Bihar, India, where it is up to 65% [11].

Other drugs, such as amphotericin B (Fungizone), liposomal amphotericin B (AmBisome), and miltefosine (Impavido), are available for the treatment of VL but are not optimal due to problems of toxicity, high price, or difficulty in administration.

### Amphotericin B

This drug is highly efficacious but is associated with serious side effects and can only be given in a hospital setting.

### Liposomal amphotericin B

This is considered to be the most effective of currently available anti-leishmanial drugs, but it is prohibitively expensive and has to be administered intravenously, making treatment more difficult under field conditions. However, recent clinical studies in India involving 203 patients showed that liposomal amphotericin B could be used as a single-dose treatment regime with a cure rate of 90% [12]. Such data are needed from African endemic areas, as it might be that response to liposomal amphotericin B can vary from species to species and in different populations. The varying doses of liposomal amphotericin B needed to achieve a cure in different endemic countries needs careful attention.

### Miltefosine

This drug is effective against VL but is expensive and teratogenic [13], so it cannot be used to treat women of childbearing age. There is a theoretical risk of resistance developing quickly to it, if it is not used in combination with other drugs. Miltefosine is registered in India for first-line treatment of VL, and in Europe for treatment of VL in patients co-infected with HIV, especially in those patients unresponsive to other treatments [14].

## Developing New Drugs

Given the problems associated with the handful of currently available drugs for VL, new and improved treatments to replace or complement existing therapy are needed urgently. Drug combinations for treating VL should provide advantages of protection from parasite resistance, as well as a reduction in treatment duration and overall toxicity.

Paromomycin (also known as aminosidine), an antibiotic of the aminoglycoside family with proven anti-leishmanial activity, is a candidate drug for treatment of VL. Early clinical studies in Kenya and India [15–17] have shown that this drug is effective in the treatment of VL. The current treatment regimen for paromomycin is 21 days when used as a single agent, but could be reduced to 17 days when used in combination with sodium stibogluconate, as field experience of Médecins Sans Frontières has shown (unpublished data).

The Drugs for Neglected Diseases Initiative is currently carrying out phase III clinical trials of paromomycin in east Africa with a view to registering the drug in Ethiopia, Sudan, and Kenya. The Institute for OneWorld Health, a nonprofit pharmaceutical company, has conducted phase III trials of paromomycin and is pursuing registration in India (see http://www.oneworldhealth.org/diseases/leishmaniasis.php).

## Vaccines: Progress and Frustrations

Extensive studies on the mechanisms of immuno-pathogenesis and protective immunity against leishmaniasis, especially in mice, have identified *Leishmania* species as good candidates for vaccine development. *Leishmania* species rarely undergo antigenic variation and show extensive cross-reactivity between different species [18,19]. Furthermore, the observation that strong lifelong immunity follows after recovery from *Leishmania* infections in humans has provided a rationale for designing immuno-prophylactic strategies against leishmaniasis. It is now a well-established fact that protective immunity to leishmaniasis is a function of cell-mediated immunity mediated by Type 1 T helper cells.

Vaccination against cutaneous leishmaniasis (CL) was a common traditional practice in the Middle East and the Soviet Union [20]. In this practice, scratched tissue from active lesions of patients with CL was applied to—or sandflies were allowed to bite—the skin of healthy individuals. Modern approaches of vaccination began by intradermal inoculation of live *Leishmania* in healthy individuals, in an attempt to produce mild, self-healing cutaneous lesions [21]. This process is often referred to as leishmanization. This term is also promiscuously used in the literature to describe the traditional practices described above. Self-healed cutaneous lesions induced by leishmanization usually confer protection against new infections. However, when leishmanization was applied to large populations, individuals developed complicated, severe, or persistent cutaneous lesions. Leishmanization has now been abandoned except in Uzbekistan [21].

Pioneered by Brazilian investigators, vaccination against *Leishmania* using killed preparations of the parasite stages has been attempted since the late 1930s [22]. Since then, killed-parasite preparations of various species and strains, with or without adjuvants, such as the autoclaved *Leishmania major* (ALM) + Bacille Calmette-Guérin (BCG) vaccines, have been extensively studied with variable success in Brazil, Venezuela, Colombia, Ecuador, Iran, and Sudan. Even though the first-generation vaccines were safe, efficacy data have not been convincing [23–27]. In Sudan, alum-precipitated ALM + BCG vaccine mixture was extensively studied and confirmed to be superior to ALM + BCG vaccine alone [28].

There has been little progress toward development of vaccines.

In general, first-generation vaccines, as attractive as they were, were met by disappointment from the scientific community, resulting in a shift of interest to novel approaches of vaccination using second-generation vaccines (recombinant molecules, and vaccines with live vectors encoding leishmanial antigens and sandfly salivary immunomodulators) [29]. Second-generation vaccines are still under development [30] with a number of ongoing safety and immunogenicity studies, but efficacy data are not expected before the next three to five years. In spite of the strong scientific conviction that leishmaniasis is prone to control by vaccines, and the extensive vaccine research carried out so far, especially in CL, no effective vaccine has yet emerged. In particular, there has been little progress towards the development of vaccines against VL.

## The Challenges Ahead

Progress towards the discovery of an effective vaccine against leishmaniasis has become a snail's race. Therefore, control of leishmaniasis by vaccines remains only a long-term goal [31].

Many leishmaniasis experts nowadays advocate vector control, especially for areas of anthroponotic transmission. History relates that in India VL was kept under control, inadvertently, by the large-scale spraying of DDT during anti-malaria campaigns [1]. Recent initiatives of the World Health Organization aim to eliminate VL from the Indian subcontinent by house-to-house spraying of DDT and to reduce epidemic CL in Kabul by a massive provision of insecticide-treated nets. Such nets have been used to reduce transmission of anthroponotic CL in Afghanistan [32]. Personal protection against the bites of *Phlebotomus orientalis* by insecticide-treated nets was considered a feasible VL control approach in Sudan [33]. In Latin America, and even more so in southern Europe, where VL is principally maintained by the domestic dog, opinions about control of VL are divided. In Southern Europe, the situation is further compounded by the increasing incidence of adult VL that is associated with HIV co-infection.

In Africa, VL is transmitted mainly in rural areas either from a zoonotic source (in sporadic endemic areas) or human to human in secondarily anthroponotic foci. Owing to the complexity and diversity of transmission patterns, but also absence of health-care settings, control of VL in the African endemic countries will indeed be challenging. In Ethiopia, HIV co-infection in some endemic areas of VL ranges from 15%–40%, and is known to be much higher in hospitals in big cities. Significant co-infection rates are being documented in Sudan. In these countries, the surveillance of HIV co-infection in VL endemic areas has to be an integral component of national VL control programmes.

The VL endemic countries provide a unique challenge to clinical research and development. Although the parasite also occurs in poor semi-urban environments, communities of affected patients are generally remote and far from health services. Government budgets are inadequate and health ministries are overstretched with many calls on their resources. In many areas hospital facilities are absent or underdeveloped. Tools for screening and identification of patients are inadequate. Current diagnostic techniques are invasive and complicated, and require trained staff. Treatments are toxic, expensive, and difficult to administer. These limitations have constrained the improvement of access to treatment. On the other hand, treatment possibilities by single-dose regimens of liposomal amphotericin B as well as the availability of miltefosine as an oral treatment of VL may provide opportunities for the development of simplified treatment regimes.

Vector control can be a useful approach to reduce the incidence of VL. Nonetheless, this is easier said than done, given the huge amounts of funds required, as well as the absence of practical decision support systems in VL endemic areas. Aside from availability of up-to-date information on VL distribution, health policymakers and health workers should be able to carry out efficient and effective vector control programmes and to properly monitor impact [34].

## Conclusion

If we are to have an impact on the incidence of the disease and curtail the negative socio-economic consequences of VL epidemics that deter human development, the international community has to address the many issues that we have raised in this article. To achieve this complex and difficult task, substantial funding will be needed, and the task will require international cooperation for success.

**Glossary**

**Domestic zoonosis:** Infection occurring via a domestic animal, e.g., dog.

**Sylvatic zoonosis:** Infection occurring via a feral mammal, e.g., mongoose in Sudan.

**Anthroponosis:** Direct human-to-human transmission by vector without an animal reservoir.

## Abbreviations

ALM: autoclaved *Leishmania major*
BCG: Bacille Calmette-Guérin
CL: cutaneous leishmaniasis
DAT: direct agglutination test
FAST: fast agglutination screening test
GIS: Geographic Information System
VL: visceral leishmaniasis
